# Fast and Medium Tempo Resistance Training with a Low Number of Repetitions in Trained Men: Effects on Maximal Strength and Power Output

**DOI:** 10.5114/jhk/161472

**Published:** 2023-04-20

**Authors:** Wei Lu, Zonghao Du, Aiguo Zhou

**Affiliations:** 1 School of Strength and Conditioning Training, Beijing Sport University, Beijing, China.

**Keywords:** movement tempo, time under tension, maximal strength, power output

## Abstract

This study aimed to investigate the effects of high load fast and medium tempo back squats using a low number of repetitions on maximal strength and power output. Seventeen participants completed a countermovement jump test and 1-repetition maximum (1-RM) assessment before and after an eight-week intervention. All participants were randomly divided into a fast tempo (FAS: 1/0/1/0) and a medium tempo (MED: 2/0/2/0) resistance training (RT) group and performed three repetitions per set of a Smith back squat exercise with 85% 1-RM intensity. Maximal strength, jump height, peak power and force of the two groups were significantly improved (p < 0.05). In addition, peak velocity significantly increased after the intervention in the FAS group (p < 0.05), but not in the MED group (p > 0.05). A significant interaction effect between training groups was observed for jump height (F (_1, 30_) = 5.49, p = 0.026, η^2^ = 0.155). However, no significant group by time interaction effects were found between training groups for maximal strength (F (_1, 30_) = 0.11, p = 0.742, η^2^ = 0.004). Therefore, the two groups showed similar effects in maximal strength, but, compared with the MED group, FAS resistance training with low repetitions caused favorable adaptations in power output in trained men.

## Introduction

Resistance training (RT) with a different movement tempo (unintentional and intentional) can induce significant changes in muscle strength and power by altering the duration of certain phases of RT (Wilk et al., 2018). An unintentional slow tempo results from a heavy load or fatigue (i.e., increased repetition duration), while an intentional slow movement tempo could be purposefully used with light loads, and when fatigue does not influence one’s ability to control movement velocity (Trybulski et al., 2021; Wilk et al., 2021). For instance, 3/1/2/0 represents a 3-s eccentric contraction, a 1-s isometric contraction, a 2-s concentric contraction, with no pauses between each repetition. However, sufficient muscle strength is necessary to control the tempo in the concentric phase (Suchomel et al., 2019). At a given load, changing the movement tempo will result in an immediate change in time under tension (TUT) and a consequent change in total training volume, in turn affecting muscle strength and power output (Gepfert et al., 2019; Hunter et al., 2003; Keeler et al., 2001; Langer et al., 2022; Wilk et al., 2020a). The duration of all efforts in a repetition and in a set of repetitions is called TUT ( Langer et al., 2022; Wilk et al., 2018). TUT is a measure of effort performed, which includes the sum of the concentric, eccentric and isometric components of a single repetition (Schoenfeld et al., 2015). Thus, TUT can be used as an indicator of exercise volume, regardless of the number of repetitions performed (Wilk et al., 2020b). To configure training programs more adjusted to practitioners’ needs, knowing the effect of manipulating the so-called acute training variables during RT is essential. Among these variables, the voluntary movement velocity has been less studied. Therefore, research on the effect of voluntary movement velocity is essential to know how to configure this variable based on the proposed objectives.

TUT has a certain impact on athletic performance (Wilk et al., 2020c). Iodice et al. (2020) compared fast tempo (FAS; 80% 1-repetition maximum (1-RM); 1/0/1/0 with 8 repetitions per set) and slow tempo (SLO; 50% 1-RM; 3/0/3/0 and repeated to failure) bilateral leg curl and leg extension exercises. Those authors concluded that the SLO group induced a specific profile of rapid muscle contraction capacity in high-level athletes and hypothesized that this phenomenon might be due to muscle deoxygenation and early recruitment of fast-twitch muscle fibers. In that study, participants in the SLO group were asked to exercise until failure, which may have led to the muscle strength gains due to muscle hypertrophy after the intervention, but the authors did not measure changes in the muscle cross-sectional area in participants. However, Wilk et al. (2021) pointed out that the most appropriate TUT for strength gain should be between 2 and 20 s per set, and Vieira et al. (2021) concluded that RT not until failure may induce comparable or even greater improvements in maximal dynamic strength and power output. González-Badillo et al. (2014) indicated that completing exercise repetitions at maximum concentric tempo for a given load could lead to greater improvements in maximal strength and power than repetitions completed at relatively slower tempos with less intent. Therefore, the effect of whether RT is repeated to failure and the association between a movement tempo and TUT on muscle strength and power remains controversial. In addition, it is necessary to be clear about the relationship between external loads and the velocity of movement. Regardless of the tempo of movement, the barbell velocity could decelerate as the external load increases (Trybulski et al., 2022). An increase in the external load likewise decreases the velocity of movement, even when the movement tempo is intentional. However, as the external load continues to increase, the difference in the velocity of movement between the volitional and maximal movement tempo decreases. The authors also found that in the squat exercise, the difference in TUT between the volitional and maximal movement tempo was constant for all external loads used (40% and 90% 1-RM), whereas in the bench press exercise, TUT increased with progressive loads. Therefore, the velocity of movement and TUT are related to the external load, movement tempo, and the type of exercise used (Trybulski et al., 2022).

The present research aimed to clarify the influence of a different movement tempo on maximal strength and power output at low repetitions. The number of sets and repetitions, interset rests and intensity were identical in both intervention groups, which only differed in the movement tempo (and consequently in TUT). Fast tempo RT (FAS) and medium tempo RT (MED) was performed three times per week for 8 weeks using the Smith back squat exercise. In addition, we hypothesized that the FAS group could achieve greater improvements than the MED group in power output, although the MED group might be slightly better than the FAS group in improving maximal strength.

## Methods

### Experimental Approach

Participants were randomly assigned to a FAS (n = 8) or a MED (n = 9) group. Before all testing and training sessions, participants were supervised during a standardized warm-up, consisting of 5 min of stationary cycling (Wattbike, West Bridgford, United Kingdom; 60 rpm, 60 W), followed by additional 8 min of self-prescribed dynamic stretching and mobility work. All tests were conducted at 9:00 am. Each participant underwent two testing sessions before and after training. The squat 1-RM was retested in the fourth week and the intensity adjusted for subsequent interventions. On the first testing day, participants performed the countermovement jump (CMJ) test after recording their anthropometric measurements. On the second testing day (after 24 h), participants performed the Smith back squat 1-RM test. After 8 weeks, all assessments were performed 72 h after the last session, respecting the same order and time intervals.

### Participants

Seventeen healthy resistance-trained men, having training experience of maximal strength, were selected (age = 18.5 ± 0.5 years; body mass = 72.1 ± 6.2 kg; body height = 179.8 ± 3.4 cm; RT experience = 5.3 ± 0.4 years; 1-RM = 131.9 ± 15.0 kg; 1-RM/body mass = 1.8 ± 0.2 kg). Participants could control the tempo with low repetitions. All participants voluntarily took part in this experiment, completed all training sessions and tests in accordance with the experimental process, and provided written informed consent. The study was approved by the Ethics Commission of the Beijing Sport University (12/2021), and all procedures were in accordance with the ethical standards of the Declaration of Helsinki, 1983.

### Procedures

#### Familiarization Session and the 1-RM Strength Test

Two weeks before the main experiment, participants completed a familiarization session. A week before the main experiment, the 1-RM squat test was conducted. Participants kept arriving at the lab at the same time each day to prepare for the upcoming experimental session (between 9:00 and 12:00 am). Upon arrival, after measuring body mass, participants warmed up on a treadmill for 5 min at an intensity of around 130 heart rate per min, followed by 10 body mass squats and eight body mass split squats (eight on each side). Subsequently, participants performed 15 to 10 repetitions of the squats at 20% and 40% of the 1-RM load, respectively. Familiarization sessions were conducted during the main testing period to minimise possible learning effects. After the warm-up, the familiarisation session began. This phase consisted of three sets of three repetitions of squat exercises, with loads reaching 80% of their estimated 1-RM.

Maximal strength was determined by assessing the 1-RM for the parallel squat (Baechle and Earle, 2008). The Smith machine was used for 1-RM testing. On the day of the test, participants warmed up with eight repetitions of 40% to 50% of the estimated 1-RM. After a 60-s rest interval, they repeated six repetitions at 50% to 60% of the estimated 1-RM. Each participant then had a maximum of five attempts to reach their 1-RM load. Participants took a 5-min rest interval between attempts. The range of motion of the squat exercises was controlled, during which participants had to reach 90° flexion (0° full extension) of the knee at the end of the eccentric phase and return to a fully extended knee position at the end of the concentric phase. Participants were verbally encouraged throughout the test, and the same researcher performed all test procedures. The test-retest reliability coefficient (ICC) was 0.92 for the squat 1-RM test.

#### Power Output

The CMJ test was performed on a Kistler force plate (Kistler Instruments, Hampshire, UK) to obtain jump height and peak power, force, and velocity. Jump height was calculated based on the participant’s flight time. The participant stood in the middle of the force plate, placed his hands on the hip joints, descended quickly, and immediately applied an upward force to jump. Participants had to jump three times in total, with an interval of ≥10 s. The highest jump height from the three attempts was selected for further analysis. Test-retest reliability coefficients (ICC) were 0.75, 0.94, 0.96, and 0.98 for jump height, peak velocity, force, and power, respectively.

### Training Protocol

After the warm-up, participants in both groups simultaneously performed 85% 1-RM back squat exercises on the Smith machine. All participants completed three RT sessions per week, for 8 weeks with five sets of three repetitions per training session. The pre-experimental back squat 1-RM test informed 85% of the 1-RM back squat performed at the start of the intervention. The training volume is shown in Table 1. The rest interval between each set was 3 min. The FAS group performed fast tempo (1/0/1/0, 1-s eccentric phase and 1-s concentric phase) and the MED group performed medium tempo (2/0/2/0, 2-s eccentric phase and 2-s concentric phase) training. Each training session was approximately ≥48 h. Participants were required to perform repetitions at an approximately constant tempo and frequency under a metronome’s tempo; participants did not perform repetitions to failure in each set. The two groups had to exercise according to the prescribed movement tempo. This was accomplished using a linear velocity transducer. A computer screen in front of participants allowed them to receive real-time auditory and visual velocity feedback. All sessions took place under investigators’ supervision, at the same time of the day (±1 h) for each participant and under constant environmental conditions (20°C, 60% humidity) (González-Badillo et al., 2014). Participants in both groups performed barbell squats with knee angles ranging from 0° to 100° flexion. During the tests, the bar was placed on a rack at a height that had to flex their knee to 100° (measured with a standard goniometer) to reach under the bar. Each repetition was videotaped to provide a sagittal view of the participant. Participants were asked not to perform additional RT during the experiment.

**Table 1:** The effect of the movement tempo on training volume.

	MED (2/0/2/0)	FAS (1/0/1/0)
% 1-RM	85%	85%
Total training session (d)	24	24
Number of sets (n)	5	5
Number of repetitions (n)	3	3
TUT per repetitions (s)	4	2
Set × Rep (n)	15	15
Set × Rep × TUT (s)	60	30
Set × Rep × TUT × Session (s)	1440	720

*FAS = fast tempo resistance training group; MED = medium tempo resistance training group; RM = repetition maximum; TUT = time under tension*.

### Statistical Analysis

Data were presented as mean ± *SD* and analyzed using SPSS 26.0 (Chicago, IL, USA), with α = 0.05 as the α level of significance. Normality tests were performed on experimental data using the Shapiro-Wilk test. Independent-samples *t*-tests were completed to examine intergroup differences. Paired-samples *t*-tests were used for intragroup comparisons. Two-way mixed analysis of variance, with Bonferroni post hoc comparisons, using one interfactor (FAS vs. MED) and one intrafactor (pre-training vs. post-training), was conducted to examine the differences across the back squat 1-RM, jump height, peak velocity, peak force, and peak power. The intraclass correlation coefficient (ICC) was used to measure the intrarater reliability. The partial Eta squared value (*η*^2^) was 0.01–0.059 for small effect, 0.06–0.137 for medium effect, >0.137 for large effect (Fritz et al., 2012). The Hedge's g effect sizes showed the difference in the relative changes from PRE to POST within-group to verify the magnitude of the difference. The cutoff values of small effect, medium effect and large effect were <0.3, 0.3–0.8, and >0.8, respectively (Turner and Bernard, 2006).

## Results

### Pretesting

All 17 participants completed the 8-week intervention; no one withdrew or suffered injury. The two groups were homogenous at baseline and showed no significant differences.

### One Repetition Maximum

**Figure 1:**
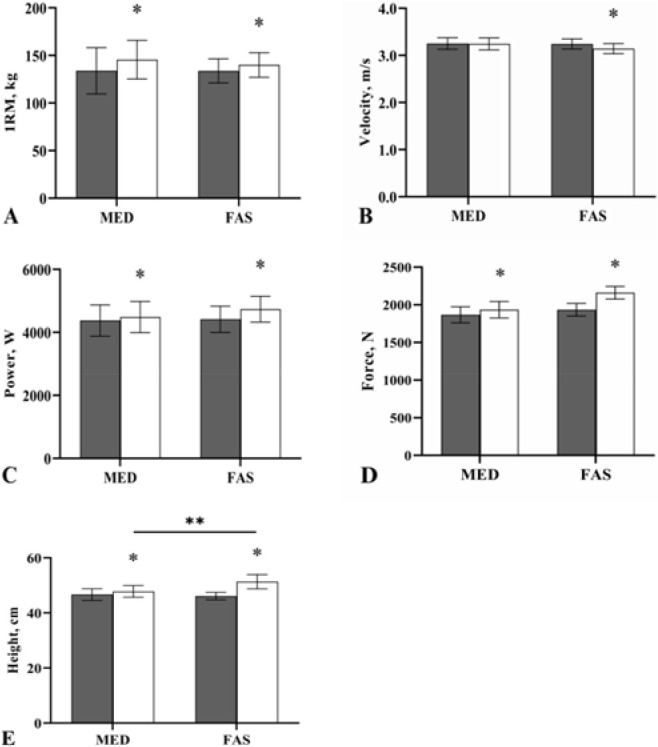
Mean difference in 1-RM (A), peak velocity (B), peak power (C), peak force (D) and countermovement jump height (E) from pre-intervention (black) to post-intervention (white) for each group. 1-RM, 1-repetition maximum; FAS: fast tempo resistance training group; MED: medium tempo resistance training group. * Significant within-group difference. ** Significant between-group difference.

Table 2 shows significant increases in maximal strength for the back squat in both groups (*p* < 0.05). Furthermore, the difference in maximal strength in the MED group (8.7%) was greater than that in the FAS group (4.7%). However, no significant group by time interaction effects were observed between training groups for the back squat (*F* (_1, 30_) = 0.11, *p* = 0.742, *η*^2^ = 0.004) (Figure 1A).

**Table 2:** Pre and post training values of different variables for fast and medium tempo resistance training groups.

	MED			FAS		
	Pre	Post	Diff (%)	Pre	Post	Diff (%)
Maximal strength (kg)	133.89 ± 31.60	145.56 ± 26.51*	8.7	133.75 ± 15.06	140.00 ± 15.41*	4.7
Peak force (N)	1867.87 ± 319.45	1935.53 ± 330.11*	3.6	1934.83 ± 239.38	2060.69 ± 240.41*	11.7
Peak power (W)	4375.21 ± 644.75	4487.26 ± 642.68*	2.6	4415.09 ± 501.05	4735.11 ± 490.37*	7.2
Peak velocity (m/s)	3.25 ± 0.16	3.24 ± 0.17	0.3	3.24 ± 0.13	3.14 ± 0.13*	3.2
Jump height (cm)	46.66 ± 2.79	47.59 ± 2.80*	1.9	46.13 ± 1.64	51.34 ± 3.12*	11.3^†^

Data are mean ± SD. FAS: fast tempo resistance training group; MED: medium tempo resistance training group. * Significant difference pre vs. post. ^†^ Significant difference MED vs. FAS.

The relative difference effect size of the PRE and POST within-group changes showed a medium effect (g = 0.38) in the MED and (g = 0.39) in the FAS group.

### Power Output

Table 2 shows that training resulted in significant increases in jump height (FAS 11.3%; MED 1.9%), peak power (FAS 7.2%; MED 2.6%), and peak force (FAS 11.7%; MED 3.6%). Furthermore, peak velocity (3.2%) significantly increased after the intervention in the FAS group (*p* < 0.05), but not in the MED group (Table 2). No significant group by time interaction effects were observed between training groups for peak velocity (*F* (_1, 30_) = 0.83, *p* = 0.371, *η*^2^ = 0.027), peak power (*F* (_1, 30_) = 0.27, *p* = 0.605, *η*^2^ = 0.009), or peak force (*F* (_1, 30_) = 0.64, *p* = 0.431, *η*^2^ = 0.021) (Figure 1B, C, and D, respectively). A significant group by time effect was recorded between groups for jump height (*F* (_1, 30_) = 5.49, *p* = 0.026, *η*^2^ = 0.155), indicating a significantly greater increase in power output after the FAS compared with the MED intervention (Figure 1E).

The relative difference effect size of the PRE and POST within-group changes for peak force, peak power, peak velocity, and jump height showed a small effect (g = 0.20), a small effect (g = 0.17), a small effect (g = 0.06) and a medium effect (g = 0.31) in the MED group and a medium effect (g = 0.50), a medium effect (g = 0.61), a medium effect (g = 0.73) and a large effect (g = 1.98) in the FAS group, respectively.

## Discussion

The present research aimed to investigate the effects of high load fast and medium tempo back squat exercise with low repetitions and relative short TUT (MED: 1440 s; FAS: 720 s) on maximal strength and power output in trained men. The main finding was that after eight weeks of Smith squat exercise, significant increases in jump height, peak power and force were observed in both the FAS and MED groups, with significant within-group increases in peak velocity occurring only in the FAS group, although for each power variable only jump height exhibited a significant group by time interaction. In addition, there was no significant interaction between groups considering the training effect in maximal strength; however, both groups showed remarkable within-group increases. Thus, both groups significantly improved maximal strength, and the FAS group presented greater improvements in power output than the MED group.

The results of this study showed a significant increase in maximal strength after training in both FAS and MED groups, but without a significant interaction between groups. Watanabe et al.’s studies showed similar results to the current research (Watanabe et al., 2013, 2014). Watanabe et al. (2013) compared the effects of fast tempo (FAS; 50% 1-RM; 1/0/1/1) and medium tempo (MED; 50% 1-RM; 3/1/3/0) knee extension exercise on muscle hypertrophy and maximal strength. After 12 weeks, the MED group showed a significant increase in muscle hypertrophy, but without significant differences in strength between groups. Subsequently, Watanabe et al. (2014) used the same movement tempo at 30% 1-RM to explore the effect on muscle hypertrophy and muscle strength, with similar results to previous research. The lack of significant differences in muscle strength between groups may be due to the relatively small changes in the movement tempo (Wilk et al., 2021). However, the difference between the concentric and eccentric contraction velocities of the two groups was only 2–3 s, which still resulted in different amplitude of the muscle strength increase (Wilk et al., 2021). On the other hand, all of these exercises involved a fairly high number of repetitions per set (from 6 to the maximum possible number of repetitions until failure), and 30% to 80% 1-RM, which may not necessarily represent optimal RT practices for improving maximal dynamic strength (American College of Sports Medicine, 2009). The interaction between different acute training variables and muscle strength remains controversial, considering that differences in intensity (Hay et al., 1983), the number of repetitions, exercise selection, and the movement tempo (Wilk et al., 2021) may all affect maximal strength. The present findings show that after an 8-week intervention, a low number of repetitions with a high load in both FAS and MED groups can improve muscle strength. Moreover, the MED group showed a higher improvement rate, which may be related to the relatively slow eccentric contraction tempo (TUT-E). A previous study has shown that longer TUT-E indirectly affects strength gains by increasing metabolic stress, hormonal responses, and muscle tension (Burd et al., 2012). Furthermore, Dankel et al. (2017) showed that variation in intensity was largely driven by specificity and neural adaptation. Meanwhile, Pereira et al. (2016) compared the effects of fast tempo (FAS; 1/0/1/0; 8-RM) and slow tempo (SLO; 4/0/1/0; 8-RM) RT on muscle strength and hypertrophy during a 12-week period of biceps curling exercises. After the intervention, the SLO group presented a significant improvement in maximal strength and hypertrophy. Therefore, one of the reasons why maximal strength improvement was higher in the MED group may be the slightly longer TUT-E, but given the relatively small difference in the movement tempo between groups, there may have not been a significant interaction.

The present study indicated that following the intervention, the FAS group achieved greater improvements in power output than the MED group as shown by the significant increase in jump height. Shibata et al. (2021) compared the effects of RT with momentary failure with different eccentric phase duration on power output, in groups carrying out fast tempo (FAS; 75% 1-RM; 2/0/2/0) and slow tempo (SLO; 75% 1-RM; 4/0/2/0) RT. After six weeks of the back squat exercise intervention, the FAS group showed significant improvements in power; decelerating the eccentric tempo did not produce better training results than in the fast eccentric group. Those results are similar to the present conclusions, except that repetitions account for higher intensities. Considering the training intensity, Cormie et al. (2010) arranged a ballistic (jump squats; 5–7 × 5–6 at 0%–30% 1-RM) and a traditional RT group (squats; 3 × 3–5 at 75%– 90% 1-RM). After the 10-week intervention, although power output improved in both groups, a significant maximal strength improvement was only observed in the traditional RT group. This suggests that high load strength training can render similar short-term improvements in athletic performance as ballistic power exercise, but with improved maximal strength. Furthermore, increased power output after high load training might be due to the increased rate of force development, neural activation rates, and intermuscular coordination, resulting in the ability to accelerate their mass to a greater degree in the same period (Cormie et al., 2011). In addition to intensity and the eccentric tempo, set length may result in performance decline (e.g., force, velocity, etc.) (Suchomel et al., 2018).

This study has certain limitations which should be addressed. The current data showed a greater difference in power indicators in the FAS than in the MED group. However, the lack of physiological and biomechanical assessment makes it hard to determine the cause of these changes. According to a recent study by Wilk et al. (2020d), we did not perform the 1-RM test independently for each movement tempo. In addition, the exercise selected for this study were squats at 85% of 1-RM, thus further research is needed to investigate the effects of a small number of repetitions when the movement tempo changes, or multi-joint exercises are used instead of single-joint exercises.

## Conclusions

During an eight-week training period, both RT protocols produced significant strength improvements, but the FAS group produced better gains in power output. The present research found that different TUT (MED: 1440 s; FAS: 720 s) corresponded to specific training effects. It is suggested that TUT should be taken into account when analyzing the volume of exercise as well as the adaptation following strength training. It is important to note that an essential skill for strength and conditioning practitioners is the ability to effectively make training adjustments to reduce the frequency and severity of injuries, overtraining, and to optimise the rate and magnitude of adaptation (Helms et al., 2020). Thus, the movement tempo and TUT need to be adjusted individually before and during training. This training protocol may not be suitable for novices due to its high load and a fast movement tempo. Therefore, our preliminary results indicate that high load fast tempo RT with a low number of repetitions can render short-term improvements in power output, as well as potential long-term benefits of improved maximal strength, which may provide significant practical implications for athletes and coaches.

## Author Contributions

Conceptualization: W.L., Z.D. and A.Z.; methodology: W.L. and A.Z.; formal analysis: W.L. and Z.D.; investigation: W.L. and Z.D.; resources: W.L. and Z.D.; writing—original draft preparation: W.L.; writing—review & editing: W.L., Z.D. and A.Z. All authors have read and agreed to the published version of the manuscript.

## ORCID iD

Wei Lu: 0000-0001-6520-9647
